# New Insights into Immunotherapy for Gynecological Cancer

**DOI:** 10.3390/jcm11144198

**Published:** 2022-07-20

**Authors:** Takuro Kobori

**Affiliations:** Laboratory of Clinical Pharmaceutics, Faculty of Pharmacy, Osaka Ohtani University, Tondabayashi 584-8540, Japan; koboritaku@osaka-ohtani.ac.jp; Tel.: +81-721-24-9374

Gynecologic malignancies are a heterogeneous group of female reproductive system tumors, including cervical, endometrial, ovarian, vaginal, and vulval cancers, and are the second most commonly diagnosed female cancers around the world [1]. Most patients with gynecological cancers in early stages are cured with surgery alone, or a combination of surgery, radiation, and chemotherapy, with excellent prognosis [2,3,4,5]. However, patients with advanced, recurrent, or metastatic diseases lack therapeutic options and often have poor survival outcomes, despite appropriate management [3,5,6,7,8,9]. Thus, there remains an urgent need to develop new therapeutic approaches for gynecological cancers.

Over the past decade, the great successes of cancer immunotherapy, especially with immune checkpoint inhibitors targeting immune checkpoint molecules such as programmed death-1 (PD-1) and PD ligand-1 (PD-L1), have fundamentally transformed the treatment landscape in a number of advanced cancers, leading to long-term survival benefits [10,11]. Patients with advanced cancers who previously had limited treatment options may now benefit from immunotherapies that offer long-lasting responses and improved survival outcomes. However, a small subset of cancer patients derives clinical benefit from immunotherapies due to intrinsic unresponsiveness and/or acquired resistance [12,13]. In particular, patients with advanced, recurrent, and/or metastatic gynecological cancers often respond poorly to immunotherapies [4,14,15,16,17,18,19,20,21,22]; thus, continued advancement in the treatment of gynecologic cancers is critical for overcoming challenges and maximizing treatment efficacy. Fortunately, exciting works to develop novel immunotherapeutic strategies against gynecological cancers are on the horizon. These represent the aim of this Special Issue in the *Journal of Clinical Medicine*, focusing on “New Insights into Immunotherapy for Gynecological Cancer” and presenting a series of four papers (three original articles and one perspective) submitted by international leaders and young researchers in this field.

Tanaka C. and colleagues opened the issue and investigated the role of ezrin/radixin/moesin (ERM) family proteins, which have a high degree of sequence similarity with each other that crosslinks several cancer-related transmembrane proteins [23,24,25,26] with the actin cytoskeleton in the plasma membrane localization of PD-L1, utilizing HEC-151 cells derived from the human endometrial adenocarcinoma [27]. Tanaka C. et al. discovered that all three ERM proteins were highly co-localized with PD-L1 in the surface plasma membrane of HEC-151 cells assessed via immunofluorescent staining with confocal laser scanning microscopy (CLSM), and that they interacted with PD-L1, as demonstrated by the immunoprecipitation assay using anti-PD-L1 antibody [27]. Interestingly, RNA interference (RNAi)-mediated knockdown of ezrin—but not radixin and moesin—significantly decreased the cell surface expression of PD-L1 without influencing its transcriptional process, highlighting the novel function of ezrin to post-translationally regulate the plasma membrane localization of PD-L1 in human endometrial adenocarcinoma cells [27]. Similarly, Tameishi M. and Ishikawa H. et al. found that ezrin also contributes to the cell surface plasma membrane localization of PD-L1 in the human epithelial ovarian cancer cell line A2780 cells [28]. Subsequently, Doukuni R. and colleagues demonstrated that in the human cervical squamous cell carcinoma (SCC) BOKU and HCS-2 cell lines, plasma membrane co-localization and protein–protein interaction of PD-L1 takes place with all three ERM proteins by means of immunofluorescence microscopy or co-immunoprecipitation experiments using a specific antibody against PD-L1, respectively [29]. Intriguingly, Doukuni R. et al. discovered that RNAi-mediated knockdown of moesin—but not ezrin and radixin—significantly reduced the cell surface expression of PD-L1 with little impact on its mRNA expression, illuminating the unexpected role of moesin in the plasma membrane localization of PD-L1, possibly via post-translational modification process, as it served as a scaffold protein in the human cervical SCC [29]. Furthermore, they also showed a higher expression of PD-L1 in the tumor samples from patients with cervical SCC and a lower survival probability in the cervical SCC patients who had high moesin expression than those who had low/medium moesin expression. This was achieved using data from The Cancer Genome Atlas, partly supporting their idea that there is a possible correlation between PD-L1 and moesin in uterine cervical SCC [29].

An excellent perspective provided by Dr. Rizzo A. offered an overview of the clinical development of immunotherapy in advanced or recurrent endometrial cancers, discussing the role of DNA mismatch repair and the “elective affinities” between immune checkpoint inhibitors and DNA mismatch repair status as a predictive biomarker in this setting [30].

This Special Issue of the *Journal of Clinical Medicine* highlighted the recent clinical advances in cancer immunotherapy for gynecological cancers and the basic science needed to explore the druggable potential therapeutic targets to regulate the expression and functionality of immune checkpoint molecules such as PD-1/PD-L1 axis in gynecological cancers, with the aim of improving the current cancer immunotherapies. Continued basic, translational, and clinical research holds enormous promise for changing the landscape of cancer immunotherapy for gynecologic cancers, which in turn may enhance the outcomes of all women suffering from these diseases.
